# Characterization of Juvenile Hormone Related Genes Regulating Cantharidin Biosynthesis in *Epicauta chinensis*

**DOI:** 10.1038/s41598-017-02393-w

**Published:** 2017-05-23

**Authors:** Ming Jiang, Shumin Lü, Yalin Zhang

**Affiliations:** Key Laboratory of Plant Protection Resources and Pest Management, National Ministry of Education, Northwest A&F University, Yangling, Shaanxi 712100 China

## Abstract

Cantharidin is a defensive toxin biosynthesized by blister beetles. It is well known for medical applications and toxicity. However, the biosynthesis process of cantharidin is still not well understood. In the present study, three genes (methyl farnesoate epoxidase (*EcMFE*), juvenile hormone acid O-methyltransferase (*EcJHAMT*) and juvenile hormone epoxide hydrolase (*EcJHEH*)) were identified from *Epicauta chinensis*. The temporal and spatial expression patterns of these three genes revealed that the expression levels of *EcMFE* and *EcJHEH* were high in the first instar larval stage of *E. chinensis* with *EcJHEH* transcripts highest in the fifth larval instar. The expression level of *EcJHAMT* was significantly higher in the 2^nd^ and 3^rd^ larval instars. The transcripts of *EcMFE*, *EcJHEH* and *EcJHAMT* showed a similar tendency with the cantharidin production in male blister beetles after mating. We verified the functions of these three genes in cantharidin biosynthesis using the RNA interference method. Interference of *EcMFE* and *EcJHEH* significantly inhibited the biosynthesis of cantharidin in male *E. chinensis* after mating, but interference of *EcJHAMT* has no apparent influence on cantharidin biosynthesis. We propose that *EcMFE* and *EcJHEH* may be involved in the biosynthesis of cantharidin, but JH III might not be the direct precursor of cantharidin.

## Introduction

Cantharidin is a defensive terpenoid found in blister beetles^1–5^. Cantharidin has been investigated in many fields due to its powerful effects on treating tumors, pests control and antibacterial activities^6–10^. Members of the Meloidae produce cantharidin and are usually used as the source for making medicines. In most meloid species, larvae sustainably produce cantharidin before pupation. After eclosion, males synthesize cantharidin persistently while females cannot produce cantharidin^11, 12^. During copulation, males transfer virtually all their cantharidin to females as a nuptial gift, and high amounts of cantharidin will then accumulate in the eggs during oviposition^4, 13^. Several meloid species such as *Epicauta mannerheimi*, *Meloetyplus fuscatus* and *Meloe proscarabaeus* mate more than once^14, 15^. Female *Epicauta nyassensis* may be able to discriminate the cantharidin content of males and prefer to mate with the one with more cantharidin reserve^3^. It also has been argued that cantharidin can function as a pheromone during courtship^2–5^.

Cantharidin is a monoterpene that is proposed to be constituted of two isoprene units^16^. By feeding isotope-labelled acetate and mevalonate, it was found that cantharidin is not formed by either a tail-to-tail or head-to-tail linkage of two isoprene units^12, 17^. Previous studies demonstrated that farnesol might act as an intermediate in the biosynthesis of cantharidin by cleaving the carbon skeleton of farnesol between C(1)-C(2), C(4)-C(5) and C(7)-C(8), and by an intramolecular conversion of incorporation into cantharidin^18–20^. Further evidence indicates biosynthesis of cantharidin in a blister beetle is effectively inhibited by 6-fluoromevalonate, which can inhibit juvenile hormone III (JH III) biosynthesis presumably by blocking the utilization of mevalonate (MVA)^21^. Our recent research shows further evidence that cantharidin is biosynthesized via the MVA pathway that can also synthesize JH III^22^.

JH is finally synthesized via methylation and epoxidation regulated by JH acid O-methyltransferase (JHAMT) and methyl farnesoate epoxidase (MFE), respectively. JHAMT was first identified and characterized in the silkworm *Bombyx mori* ^23^ and then in other species including the red flour beetle *Tribolium castaneum* ^24^, desert locust *Schistocerca gregaria* ^25^ and honeybee *Apis mellifella* ^26^. JHAMT protein methylates carboxyl groups in JH III acid and farnesoic acid (FA) producing JH III and methyl-farnesoate (MF), respectively^24^. MFE protein which can epoxidize MF to JH III was first identified as a microsomal cytochrome P450 enzyme in the cockroach *Diplioptera punctata* ^27^. The activity of epoxidation FA to JH III acid was demonstrated in *B. mori* ^28^. MFEs were also identified and characterized in other species, such as *T. castaneum* ^29^ and *S. gregaria* ^25^. In insects, JH III is principally degraded by JH epoxide hydrolase (JHEH) hydrolyzing JH III to JH III diol or converting JH III to JH III acid diol via JH III acid with the presence of JH esterase^30–32^. Several JHEHs have been identified and characterized in insect species such as *T. castaneum* ^33^, potato beetle *Leptinotarsa decemlineata* ^34^, *B. mrio* ^32^ and *A. mellifera* ^35^. Previous studies about cantharidin biosynthesis have provided some clues: 1) cantharidin is synthesized via a MVA pathway; 2) farnesol may be the intermediate of cantharidin biosynthesis in blister beetles; and 3) a JH metabolite is supposedly associated with the biosynthesis of cantharidin. Nonetheless, whether JH or a JH metabolite is directly involved in the biosynthesis of cantharidin remains obscure. Identifying whether the genes MFE, JHAMT and JHEH (which are related to the synthesis and metabolism of JH III) are involved in the biosynthesis of cantharidin could help us to analyze the role of JH III on cantharidin biosynthesis.

In the present study, we cloned and characterized MFE, JHAMT and JHEH genes from *Epicauta chinensis* and analyzed the temporal and spatial expression patterns of these three genes. Furthermore, the functions of these three genes on cantharidin biosynthesis *in vivo* were elucidated using RNA interference experiments.

## Results

### Identification and characterization of three genes

UTR sequences of *Epicauta chinensis* methyl farnesoate epoxidase (*EcMFE*), JH epoxide hydrolase (*EcJHEH*) and JH acid O-methyltransferase (*EcJHAMT*) were obtained by the RACE method. To verify the accuracy of sequences, we sequenced PCR products spanning the entire open reading frame (ORF) of these three genes. The full-length cDNA sequence of *EcMFE* (GenBank accession number: KX855996) is 1625 bp, and that of *EcJHEH* (KX855995) and *EcJHAMT* (KX880373) is 1473 bp and 1066 bp, respectively. Other detailed information on sequences for these three genes is shown in Table 1. Signal peptide cleavage sites were detected at 24 to 25 and 19 to 20 amino acids (aa) for EcMFE and EcJHEH, respectively. No signal peptide cleavage site was detected in the EcJHAMT amino acid sequence. EcMFE Comparisons with database entries of GenBank by using the blastp obtained 81, 61, 56, 55 and 52% amino acid identity with reported MFEs from *Tribolium castaneum* (EFA01264.1), *Nicrophorus vespilloides* (XP_017774921.1), *Agrilus planipennis* (XP_018325317.1), *Diploptera punctata* (Q6R7M4.1) and *Schistocerca gregaria* (ADV17351.1). Comparisons of EcJHEH and EcJHAMT showed the highest Bit scores to amino acid residues of *T. castaneum* JHEH (EFA00568.1) and JHAMT (BAG30999.1), with 65% and 45% identities, and 78% and 68% positives, respectively.

**Table 1:** Information of cDNA and amino acid sequences.

Gene	Full-length sequence	5′ UTR	3′ UTR	ORF	Encoding protein	Molecular mass	Theoretical isoelectric
EcMFE	1625 bp	61 bp	76 bp	1488 bp	495 aa	54.81 kDa	8.29
EcJHEH	1473 bp	39 bp	60 bp	1374 bp	457 aa	52.20 kDa	8.76
EcJHAMT	1066 bp	144 bp	92 bp	830 bp	283 aa	33.01 kDa	5.06

The analysis of deduced amino acids of *E. chinensis* MFE revealed the Cytochrome P450 conserved site was located at 432–441 aa. EcMFE was predicted to have a membrane anchor region at 2–19 aa (Figure S1A). The conserved domain search on the NCBI database revealed that the predicted ORF of EcJHEH contains an epoxide hydrolase N-terminus domain at 51–153 aa, and an alpha/beta hydrolase domain at 144–244 aa (Figure S2A). Methyltransferase and S-adenosylmethionine (SAM)-dependent methyltransferases domains were found at 41–138 aa and 39–145 aa for *E. chinensis* JHAMT. The transmembrane region was found at 141–158 aa (Figure S3A). To investigate the phylogenetic relationship of the three proteins (EcMFE, EcJHEH and EcJHAMT) and its orthologs, we constructed phylograms based on 9 MFEs (Figure S1B), 17 JHEHs (Figure S2B) and 12 JHAMTs (Figure S3B) protein sequences of insects from GenBank, respectively.

### Temporal and spatial expression patterns of three genes in *E. chinensis*

**Figure 1:**
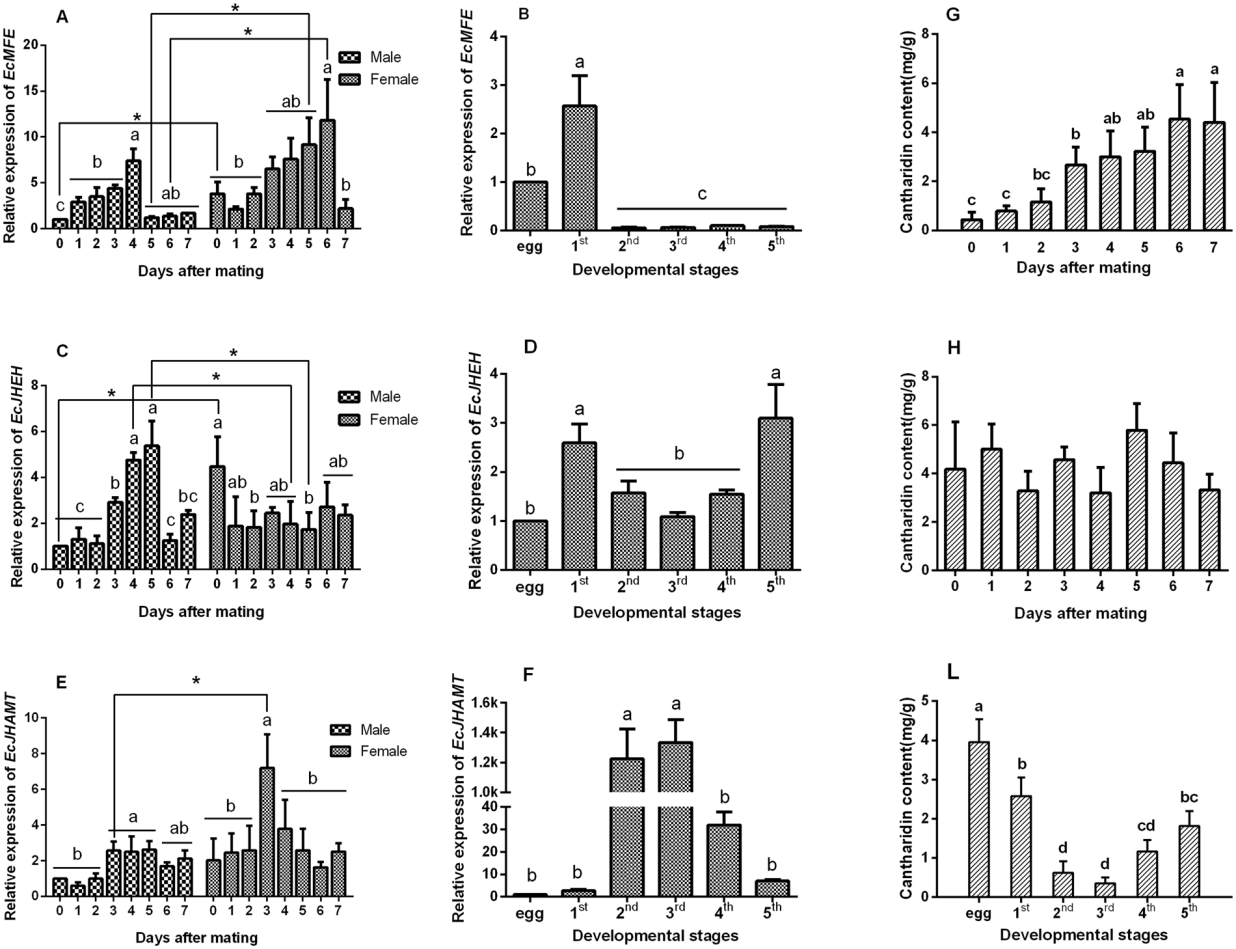
Relative expression profiles of *EcMFE*, *EcJEHE* and *EcJHAMT* mRNA and cantharidin content at developmental stages in *Epicauta chinensis*. (**A**,**C** and **E**) are expression levels on the 0 to 7^th^ day after mating in *E. chinensis*. (**B**,**D** and **F**) are expression levels in different instar larvae and eggs, (**G**,**H** and **L**) are cantharidin content at different developmental stages. Asterisks above represent statistically significant differences by Student’s *t*-test (P < 0.05), and different letters on error bars indicate significant differences in each group by one-way ANOVA analysis (P < 0.05).

In order to clarify the temporal and spatial expression patterns of these three genes in *E. chinensis*, we determined the relative expression of *EcMFE, EcJHEH* and *EcJHAMT* in eggs, different instars and adults from just-mated (0 day) to the 7^th^ day after mating and different tissues of adults 3 days after mating by quantitative real-time PCR (qRT-PCR). Results showed that the expression level of *EcMFE* increased from the 0 to 4^th^ day after mating in male blister beetles, and was maintained at a low level, the same as those just-mated from the 5^th^ to 7^th^ day after mating (Fig. 1A). There were two obvious rising periods shown on the 1^st^ and 4^th^ day after mating, respectively. The relative expression of *EcMFE* indicated the highest level was in the 1^st^ instar larva of *E. chinensis*. It was about 2.5-fold higher than in the eggs. However, expression levels of *EcMFE* were much lower from the 2^nd^ to 5^th^ instar larvae, less than 4 percent of that in the first instar larva (Fig. 1B). The expression level of *EcJHEH* increased from the 2^nd^ to 5^th^ day after mating in male blister beetles; then transcript levels on the 6^th^ and 7^th^ day decreased to the same level as in the first three days after mating (Fig. 1C). In the larval stage, *EcJHEH* expression revealed a high level in the 1^st^ and 5^th^ instar, nearly twice the level in the 3^rd^ instar which exhibited the lowest expression level in developmental stages (Fig. 1D). The results of *EcJHAMT* indicated that *EcJHAMT* transcripts were more abundant in the male adults at 3–5 days after mating than on the first three days (Fig. 1E). However, the *EcJHAMT* transcript exhibited an extremely high level in the 2^nd^ and 3^rd^ instar larvae of *E. chinensis*, over 600-fold the expression level in the first instar larva (Fig. 1F).

In female blister beetles, the *EcMFE* transcript revealed a similar tendency to that in males from 0 day to 6 days after mating. The highest expression level in females was higher than in male *E. chinensis* (Fig. 1A)*. EcMFE* and *EcJHAMT* expression levels were higher than males in female beetles, and the most abundant transcript of *EcJHAMT* showed on the 3^rd^ day after mating (Fig. 1E). The highest transcript level of *EcJHEH* was exhibited in just-mated females of *E. chinensis;* the expression level was then maintained at a low level, the same as that on the first three days after mating in male blister beetles (Fig. 1C).

**Figure 2:**
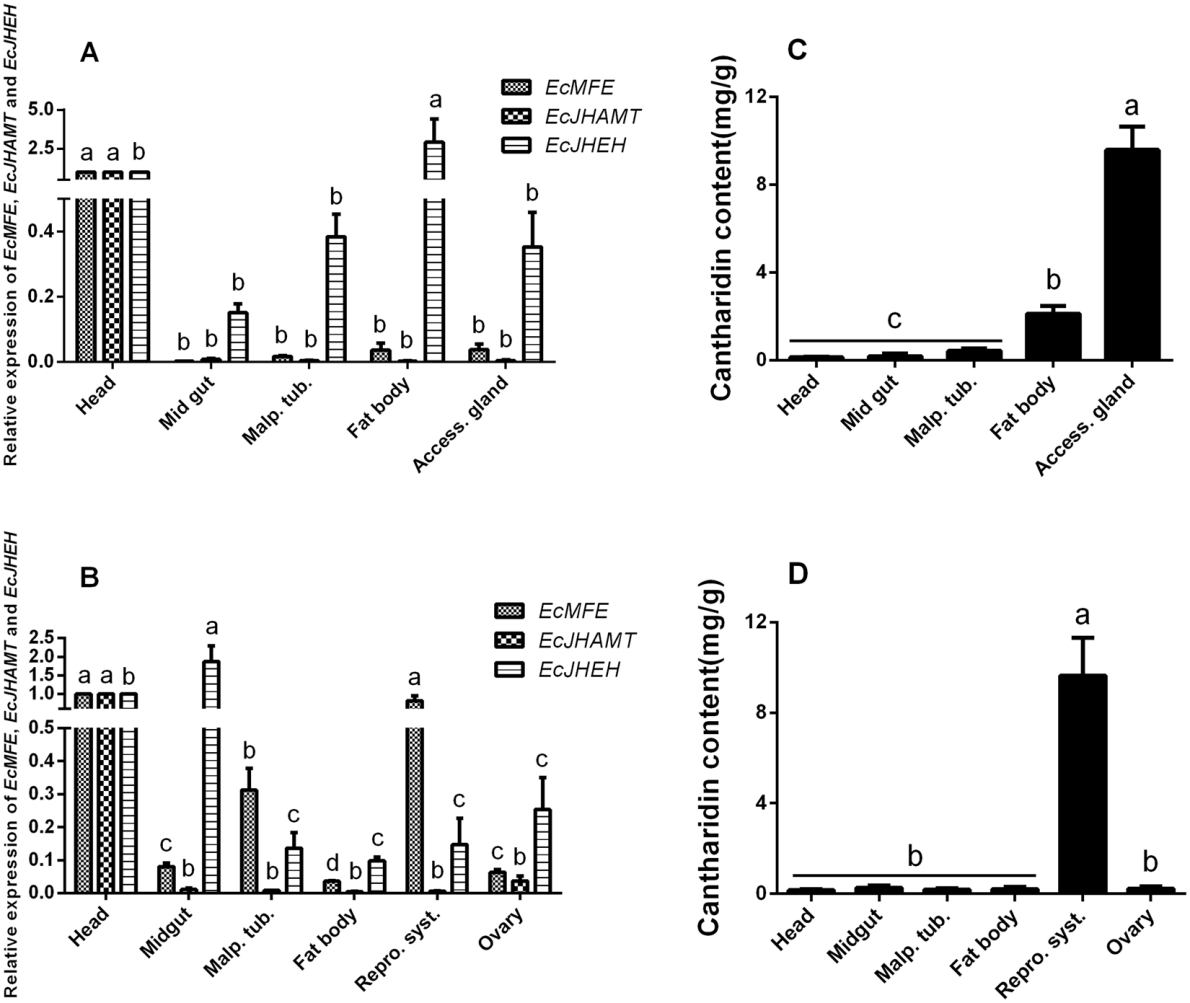
Relative expression profiles of *EcMFE*, *EcJEHE* and *EcJHAMT* mRNA and cantharidin content in different tissues from adult *E. chinensis*. (**A**) Relative expression profiles in male tissues. (**B**) Relative expression profiles in female tissues. (**C**) cantharidin content in male tissues. (**D**) cantharidin content in female tissues. Different letters on error bars indicate significant differences in each group by one-way ANOVA analysis (P < 0.05).

Further more, we determined the relative expression of *EcMFE*, *EcJHEH* and *EcJHAMT* in different tissues from adult *E. chinensis*. The results revealed that the expression levels of *EcMFE* and *EcJHAMT* were significantly high in the head of males. While the transcripts were maintained at a low level in other tissues from males. The transcript of *EcJHEH* showed the significantly high level in the fat body of male blister beetles, nearly three times the level in the head which showed the second-high level (Fig. 2A). In tissues from female *E. chinensis*, *EcJHAMT* exhibited the similar expression trend of that in males. However, *EcMFE* showed the significantly high level not only in the head but also in the reproductive system without ovary. For *EcJHEH* gene, the highest expression level appeared in the midgut (Fig. 2B).

### Cantharidin content in *E. chinensis* at different stages and in different tissues

We measured the cantharidin content of *E. chinensis* to verify the correlation the cantharidin production and the three genes transcription. During the 7 days after mating, the cantharidin content in male *E. chinensis* accumulated gradually. On the 7^th^ day after mating, the content of cantharidin reached 4.41 mg/g (dry weight) which was over 10 times higher compared to that on day 0 (Fig. 1G). In addition, Cantharidin content slowly accumulated over the first three days after mating, and then appeared to accumulate more slowly thereafter. In females, cantharidin content was maintained at approximately 4 mg/g from day 0 to day 7 after mating, with no significant difference between samples (Fig. 1H).

Furthermore, cantharidin was detectable in all the first five larval instars and eggs, with the highest relative content of 3.96 mg/g at the egg stage (Fig. 1L). Cantharidin content was lowest in the 2^nd^ and 3^rd^ instars, which was only a tenth of that in eggs, and gradually increased in the 4^th^ and 5^th^ instar larvae.

We also measured cantharidin production in various tissues from adult blister beetles. In males, the accessory gland had the highest cantharidin content, which was over 70-fold higher than that contained in head (0.12 mg/g) (Fig. 2C). Unexpectedly, the fat body showed the second highest cantharidin content (approximately 2.11 mg/g), making it significantly different from other tissues. In female blister beetles, the highest cantharidin content exhibited in reproductive system without ovary and shown an extremely significant differences with other tissues (Fig. 2D).

### Effects of RNA interference

**Figure 3:**
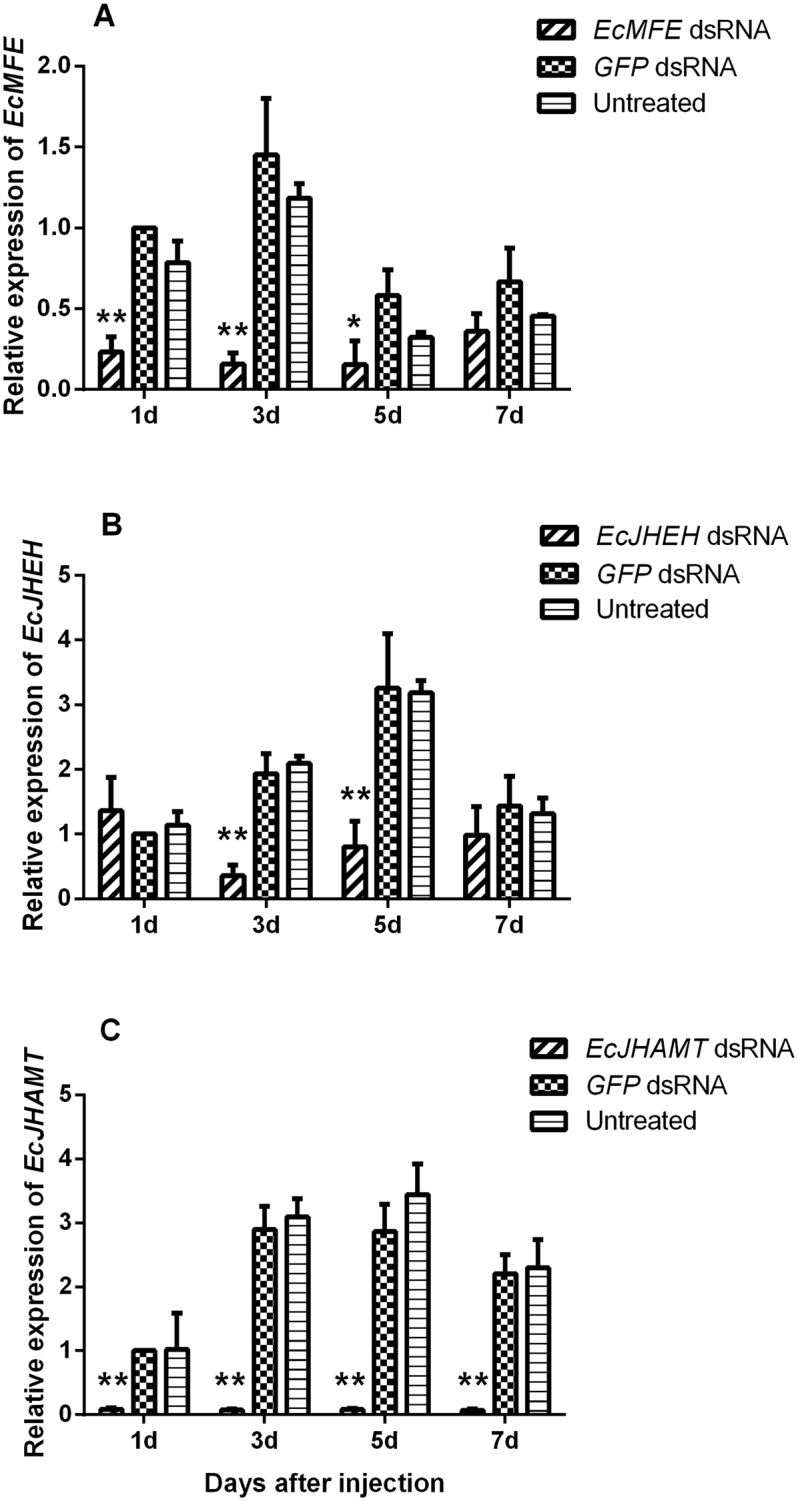
Relative expression profiles of genes in male *E. chinensis* at different times after dsRNA injection. (**A**) *EcMFE*. (**B**) *EcJHEH*. (**C**) *EcJHAMT*. Asterisks above represent statistically significant differences by Student’s *t*-test (*P < 0.05; **P < 0.01).

To clarify the function of *EcMFE*, *EcJHEH* and *EcJHAMT* on cantharidin production, we carried out RNA interference (RNAi) in just-mated male *E. chinensis*, the time window for rapid cantharidin production^22^. The efficiency of RNAi-mediated knockdown was detected by qRT-PCR. Results showed that the expression levels of *EcMFE*, *EcJHEH* and *EcJHAMT* were all significantly suppressed in treated beetles (Fig. 3). In *EcMFE* treatment, the transcript level was significantly reduced to 23 percent of the level in controls (GFP double stranded RNA (dsRNA) injected and untreated groups) just 24 h after injection. After 3 days, the transcript level was diminished to 10 percent, and was 26 percent at the 5^th^ day (Fig. 3A). RNAi in the *EcJHEH* dsRNA injection treatment only exhibited a significantly higher level on the 3^rd^ and 5^th^ day after injection, but it made the *EcJHEH* transcript stay at a rather low level compared with controls (Fig. 3B). Unexpectedly, the results of the *EcJHAMT* knockdown treatment suggested that injection of *EcJHAMT* dsRNA induced extremely sensitive systemic RNAi responses in male *E*. *chinensis* (Fig. 3C). The highest expression level in controls was over 50-fold more than that in the 5^th^ day after *EcJHAMT* dsRNA injection. These results confirm that the RNAi of these three genes was highly effective.

### Effects of RNAi-mediated knockdown on cantharidin production

The RNAi-mediated knockdown of *EcMFE*, *EcJHEH* and *EcJHAMT* was performed by injecting dsRNA into the just-mated male blister beetles. Almost all of the beetles that received *EcMFE*, *EcJHEH*, *EcJHAMT* or *GFP* dsRNA were alive through the day for cantharidin and RNA extraction. In order to determine whether the successful RNAi of these three genes would influence the synthesis of cantharidin in *E*. *chinensis*, we detected the cantharidin content as above.

**Figure 4:**
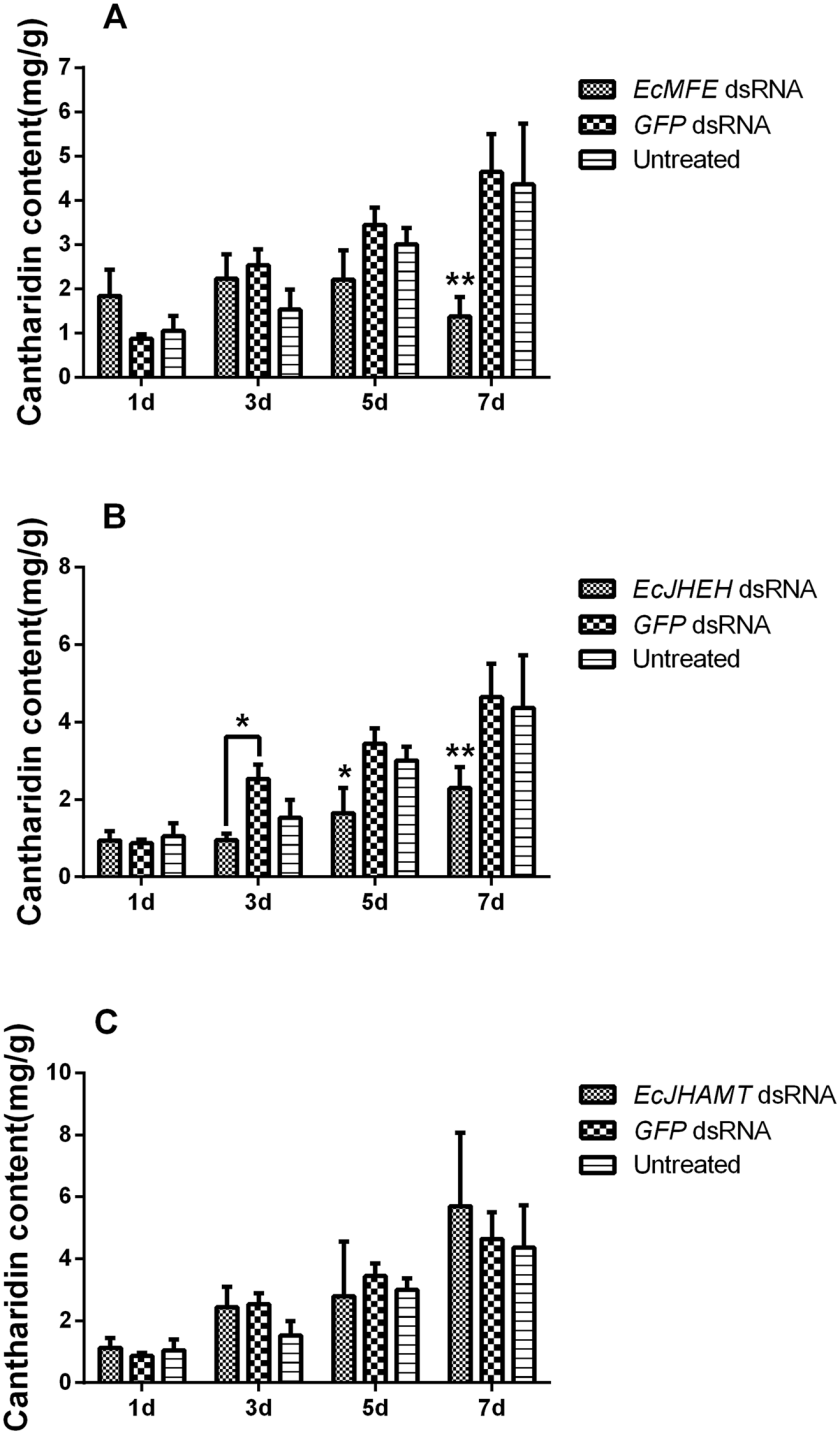
The cantharidin content in male *E. chinensis* at different times after dsRNA injection. (**A**) *EcMFE*. (**B**) *EcJHEH*. (**C**) *EcJHAMT*. Asterisks above represent statistically significant differences by Student’s *t*-test (*P < 0.05; **P ≤ 0.01).

In *EcMFE* dsRNA treatment, there were no significant differences between the treated group and controls until the 5^th^ day after injection, when compared on cantharidin content. However, the cantharidin content was significantly reduced on the 7^th^ day after injection (P < 0.01, Fig. 4A). Furthermore, we found that in beetles that received *EcJHEH* dsRNA, cantharidin production was arrested at a low level, and showed significant differences with controls beginning from the 3^rd^ day after injection (Fig. 4B). In contrast, *EcJHAMT* dsRNA injection results revealed no significant variation in cantharidin content in males compared with controls in all tested beetles (Fig. 4C). There were no differences between the two controls (GFP dsRNA injected and untreated beetles) at different times after injection.

## Discussion

We isolated and cloned methyl farnesoate epoxidase, JH acid O-methyltransferase and JH epoxide hydrolase genes from *E. chinensis*. These cDNA sequences and the deduced amino acid sequences reflect a high degree of homology with genes identified from other insects, especially *T. castaneum*. Furthermore, the function domains specific to each protein were found in the deduced protein sequence of these three genes. EcMFE has an 18 aa hydrophobic N-terminal anchor preceding a single charged residue, P450 conserved site and a typical “PPGP” hinge homologous to microsomal cytochrome P450 enzyme^36^. The analysis of EcJHAMT reveals a SAM-dependent methyltransferases domain is found at 39–145 aa, which utilizes the methyl group of SAM to methylate proteins, small molecules, lipids and nucleic acids^37^. The presence of motif (ILDIGCGDG) indicates that EcJHAMT belongs to the SAM-dependent methyltransferases superfamily, similar to previously-described JHAMT orthologs in other insects^23–26^. We analyzed the deduced amino acid sequence of EcJHEH in the NCBI data base; the results show that EcJHEH contains an epoxide hydrolase N-terminus domain at 51–153 aa, and an alpha/beta hydrolase domain at 144–244 aa. These indicate EcJHEH belongs to the α/β hydrolase superfamily and hydrolyses its substrates by the action of a catalytic triad^32, 33, 35^. From sequence analysis of EcMFE, EcJHAMT and EcJHEH, we are confident that the cDNA newly isolated in the present study encodes MFE, JHAMT and JHEH proteins in *E. chinensis*.

Cantharidin is a defensive toxin synthesized by meloid insects^11^. Previous studies have well-demonstrated cantharidin is biosynthesized via the MVA pathway and regulated by 3-hydroxy-3-methylglutaryl coenzyme-A reductase in blister beetles^19–22^. Moreover, cantharidin production varies at different stages of development in the blister beetle^2, 13, 22^. Results of cantharidin content detection show that there is a synthesis period for cantharidin in male blister beetles after mating (Fig. 1G). Nevertheless, cantharidin content is maintained at a high level with no significant difference from day 0 to day 7 after mating in females (Fig. 1H). In eggs and larval stages, cantharidin content shows the lowest level in the 2^nd^ and 3^rd^ instar larvae (Fig. 1L). In our present study, we examined temporal expression patterns of *EcMFE, EcJHAMT* and *EcJHEH* mRNA in *E. chinensis* at different developmental stages and the adults on different days after mating to determine the relationship among these genes and cantharidin biosynthesis. The qRT-PCR data revealed that the three genes show different expressional tendencies both in larval and adult blister beetles (Fig. 1A–F). Combined with cantharidin content analysis, we found that these three genes exhibited correspondence with cantharidin production in male *E. chinensis* after mating, at least in the first four days. In the larval stages of *E. chinensis*, only the *EcJHEH* mRNA transcript revealed a correspondence with the cantharidin production (Fig. 1D), whereas *EcJHAMT* shows an almost opposite relationship (Fig. 1F). JH is a developmental regulating hormone that plays a critical role in the regulation of insect metamorphosis, especially in larvae. The inconsistency of expression levels of *EcMFE* and *EcJHAMT* with cantharidin production in larvae may be due to the high synthesis of JH III in larvae of *E. chinensis* ^24, 29^. Furthermore, similar phenomena were found in female adults *EcMFE* and *EcJHAMT* transcripts were more abundance in females after mating than males but *EcJHEH* expression levels were lower. Previous research revealed that JH can regulate the reproduction and synthesis of vitellogenin in mature female insects^38–40^. Similar to the larvae, the high expression of *EcMFE* and *EcJHAMT* may be related to the high synthesis of JH in female adults. From these results, we speculated that *EcMFE* and *EcJHAMT* have a large role in JH synthesis, and the increasing expression of *EcMFE, EcJHAMT* and *EcJHEH* mRNA may be related to cantharidin production in male blister beetles after mating.

**Figure 5:**
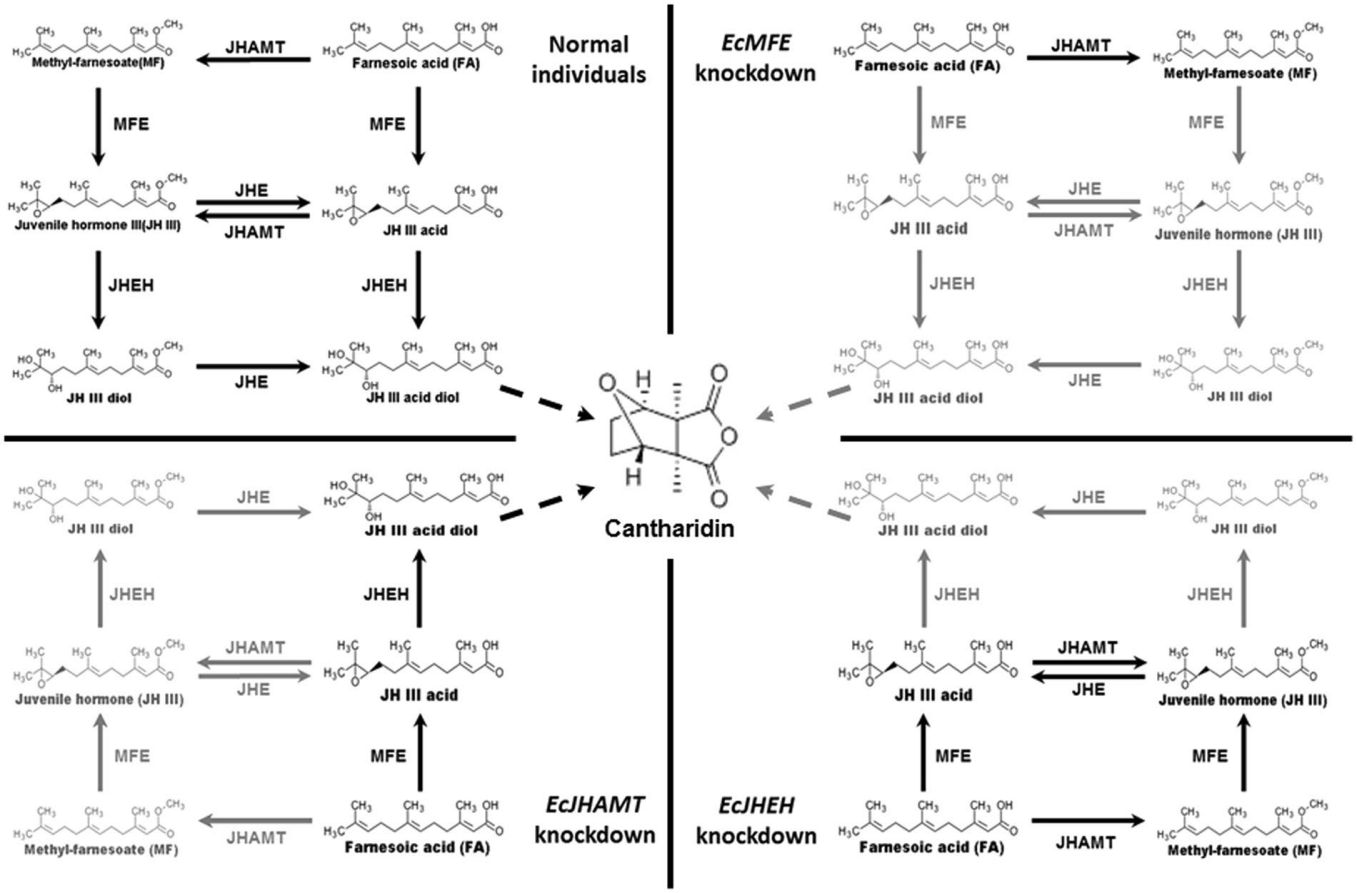
Diagram of the process of *EcMFE*, *EcJHAMT* and *EcJHEH* regulating cantharidin biosynthesis. Reactions that could occur normally are shown in black. Reactions that could not occur after dsRNA knockdown are shown in gray.

We compared the mRNA expression levels of *EcMFE*, *EcJHEH* and *EcJHAMT* and cantharidin content in different tissues of male and female *E. chinensis* on the 3^rd^ day after mating. For cantharidin content detection in different tissues from male and female *E. chinensis*, the significantly high content of cantharidin in females is in the reproductive system without ovary and the content in other tissues is maintained at an extremely low level (Fig. 2D). In males, the highest cantharidin content shows in the accessory gland. While the content in the fat body is the second highest level in males and significantly high than that in other tissues (Fig. 2C). Previous study^11^ has reported that the accessory gland of male blister beetles acts as a storage gland for cantharidin more than the site of cantharidin biosynthesis. Due to lack of the ability of the cantharidin producing in female blister beetles after eclosion^11, 12^, the high content of cantharidin in the fat body of male *E. chinensis* which different with females may relate to the biosynthesis of cantharidin in males. In males, the expression level of *EcMFE* and *EcJHAMT* show a significantly high level in the head than other tissues (Fig. 2A). While the highest transcript level of *EcJHEH* is in the fat body which contain the second-high level of cantharidin content among the tissues of male blister beetles (Fig. 2A and C). In females, the expression level of *EcJHAMT* show the same tendency with that in males, whereas *EcMFE* exhibits the similarly high level not only in the head but also in the reproductive system without ovary (Fig. 2B). The significantly high expression level of *EcJHEH* appears in the midgut of females different with that in males. The high expression level of *EcJHEH* in the fat body of male *E. chinensis* may indicate that the transcript level of *EcJHEH* and cantharidin producing have high correlativity. For *EcMFE* gene, the high expression levels exhibit in the head and reproductive system reveal that *EcMFE* transcript level may appear more relevant with the synthesis of JH III than cantharidin. Previous studies suggest that the synthesis of JH III is performed primarily by the epoxidation of FA into JH III acid before the esterification of JH III acid into JH III in Lepidoptera^29, 41^. In orthopteroid insects, esterification of FA into MF occurs before epoxidation of MF into JH III^42^. Recently research has shown that JHAMT protein in *T. castaneum* can catalyze the methylation of both FA and JH III acid to generate MF and JH III, respectively^24^. And TcCYP15A1 the methyl farnesoate epoxidase in red flour beetle epoxidizes both FA and MF to generate JH III acid and JH III, respectively^29^. Therefore it appears that there are two pathways to synthesize JH III in beetles. One is epoxidizing FA to JH III acid first, and then methylating JH III acid to JH III. The other is generating MF first and then epoxidizing it to JH III. In order to verify whether *EcMFE*, *EcJHAMT* and *EcJHEH* could regulate the biosynthesis of cantharidin in *E*. *chinensis*, the knockdown of these three genes in just-mated male adults was performed by RNA interference. Results show that the efficiencies of RNAi-mediated knockdown are significantly high in these three genes (Fig. 3). In the *EcMFE* dsRNA treatment, it shows an obviously lower cantharidin content on the 7^th^ day after injection compared with controls (Fig. 3A). This indicates that *EcMFE* is involved in the process of cantharidin biosynthesis in the male *E. chinensis* after mating. The MFE enzyme could epoxidize FA or MF into JH III acid or JH III^27, 28^. This result suggests that JH III and JH III acid might be related to cantharidin production in male blister beetles (Fig. 5). In addition, in *EcJHEH* knockdown beetles cantharidin production was significantly inhibited on the 5^th^ and 7^th^ day after injection (Fig. 3B). This indicates that *EcJHEH* is also involved in the biosynthesis of cantharidin in male blister beetles after mating. The JHEH enzyme can both hydrolyze the epoxy group of JH III and JH III acid to generate JH III diol and JH III acid diol, respectively^30–32^. Successful knockdown of *EcJHEH* shows inhibition of the synthesis of JH III diol and JH III acid diol, but may provide a relatively high content of JH III and JH III acid. These results reveal that JH III and JH III acid may be not be necessary compounds for cantharidin synthesis in male *E. chinensis* after mating. But JH III acid diol and JH III diol may play a more direct role in cantharidin production (Fig. 5). However, there was no significant difference in cantharidin production between the *EcJHAMT* knockdown beetles and controls. Previous studies indicated FA or JH III acid can be esterified into MF, or JH III can be catalyzed by JHAMT enzyme, respectively^24^. From Fig. 5 we can infer that successful *EcJHAMT* knockdown would inhibit the synthesis of JH III diol and JH III, which has no effect on cantharidin production. This further indicates that JH III and JH III diol may be not essential in cantharidin biosynthesis, and JH III acid diol may in be involved in cantharidin biosynthesis. Our previous research has demonstrated that EcHMGR can significantly regulate the biosynthesis of cantharidin in male *E. chinensis* after mating. Further experiments revealed that injecting exogenous JH III did not increase the cantharidin content in the *EcHMGR* RNAi-knockdown blister beetles. According to the above evidence taken together, we infer that JH III might not be the essential factor in cantharidin biosynthesis, while JH III acid diol could be more important in this process.

In summary, we isolated and characterized *MFE, JHAMT* and *JHEH* genes from *E. chinensis*, then examined temporal and spatial expression patterns of these three genes. Overall the mRNA expression level of *EcMFE, EcJHAMT* and *EcJHEH* increase from just-mated to the 5^th^ day after mating in male blister beetles, and show a similar tendency with cantharidin production. In larva stages, only *EcJHEH* exhibited the same tendency with cantharidin production. Furthermore, we analyzed the roles of these three genes on cantharidin biosynthesis by RNAi, and analyzed the relationship between cantharidin synthesis and JH III and its metabolite. Interference of *EcMFE* and *EcJHEH* significantly inhibited the biosynthesis of cantharidin in male *E. chinensis* after mating. In conclusion, *EcMFE* and *EcJHEH* may be involved in the biosynthesis of cantharidin, but JH III may not be the direct precursor of cantharidin.

## Materials and Methods

### Insect

Wild *E. chinensis* beetles were collected from Suide County (Shaanxi, China), during their eclosion peak. The blister beetles were reared in the laboratory with fresh Lucerne and soybean leaves at 28 °C and under a 14:10 L:D photoperiod. We isolated copulating pairs from the wild strain, selected male adults that had just finished mating, and reared them independently under the same conditions as above. Larvae of *E. chinensis* were reared in the laboratory (25 °C, 14:10 L:D and 60% RH) on eggs of *Locusta migratoria*. About 3 triungulin larva were reared in a plastic cup (5 cm diameter and 8 cm high) with several egg masses and covered with soil to avoid cannibalism. RNA and cantharidin were isolated from 5 adults and at least 10 eggs or larvae for each replicate. Tissue samples were taken from adults 3 days after mating and pooled from 20 males or females *E. chinensis*. Three independent biological replicates were carried out in all experimental series.

### RNA isolation and cDNA cloning

Total RNA was extracted using the Trizol method (TaKaRa, Japan) with the whole bodies of male *E. chinensis*. First strand cDNA was synthesized with a PrimeScript™ II 1^st^ Strand cDNA Synthesis Kit (TaKaRa) using random 6 mers primers in total volume 20 μL. 5′ and 3′ RACE reactions were performed using SMARTer RACE 5′& 3′ Kit (TaKaRa) according to the manufacturer’s instructions. The primary and nested gene-specific primers used for 5′ and 3′ RACE PCR were designed by using the RNA-sequencing database of *E. chinensis* (unpublished) and shown in Table S1. For all primer pairs, the following cycling profile was carried out in a Bio-Rad thermal cycler: 95 °C for 5 min; 37 cycles of 95 °C for 30 s, Tm °C for each pair for 30 s, 72 °C for 1 kb/min; and a final 10 min extension at 72 °C. PCR products were purified using a Gel extraction kit (Code No. BSC02S1, Biospin, China) and cloned into a pMD^TM^ 19-T vector (TaKaRa). Recombinant plasmids were subsequently purified for sequencing.

### Sequence analysis

Nucleotide sequencing was achieved commercially (AuGCT, Inc., Beijing, China). Sequences were processed with sequence analysis software V.7.0(DNASTAR Inc., Madison, WI, USA). Protein sequence similarity searches were performed using the BLASTP in GenBank (http://blast.ncbi.nlm.nih.gov/Blast.cgi). Protein sequence analysis was performed using the ExPASy proteomics tools (http://www.expasy.org/proteomics). The transmembrane segments were predicted by TMHMM (http://www.cbs.dtu.dk/services/TMHMM/). Sequence alignments were performed by Clustal Omega (http://www.ebi.ac.uk/Tools/msa/clustalo/). And the phylogenetic tree was compiled using MEGA 6.0 software.

### Double stranded RNA synthesis and injection

To produce dsRNA for RNA interference, 463 bp, 489 bp and 545 bp PCR fragments of *EcMFE, EcJHEH* and *EcJHAMT* genes respectively were amplified using cDNA described above as template and primers as shown in Table S1. Purified PCR products were used as templates to synthesize dsRNA using T7RiboMAX^TM^ Express RNAi System (Promega). A 541 bp PCR fragment from the *GFP* gene (L4440 plasmid, Addgene Inc., Cambridge, MA, USA) was used to prepare control dsRNA. The purified dsRNA was detected and stored at −80 °C until use.

dsRNA was injected into just-mated male *E. chinensis*. The insects were kept on ice for 20–30 mins prior to injection. 6 μg dsRNA was injected from the intersegmental membrane of the ventral side using an aspirator tube assembly fitted with a needle pulled from a glass capillary tube by a flaming/Brown micropipette puller (Model P-97, Sutter Instrument Company, Novato, CA, USA). Injected beetles were raised in a small chamber separately from the females. Fifteen beetles were sampled independently for each treatment for gene expression analysis and cantharidin detection on 1, 3, 5 and 7 days after injection.

### Quantitative real time PCR

Quantitative real time PCR was used to detect gene expression in different samples. RNA isolation was done as mentioned above. RT-PCR and qRT-PCR were performed using PrimeScript RT Reagent Kit with gDNA Eraser and SYBR Premix Ex Taq Kit (TaKaRa) respectively, according to the manufacturer’s instructions. The primers used for qRT-PCR were designed from the identified PCR fragments using the Primer 3 Software(http://www.simgene.com/Primer3) for the PCR product of about 100 bp in length (Table S1). PCR amplification and fluorescence detection were performed using a Cylcer iQ Real-time PCR System (Bio-Rad iQ5 Hercules, CA, USA). The protocol was applied: 10 s at 95 °C; 40 cycles of 30 s at 95 °C, 30 s at 60 °C followed by melting curve analysis. Three independent technical replicates were included for each sample. Expression of the *E. chinensis* actin gene^43^ was used as an endogenous control to normalize the expression data and the expression level of four genes were analyzed by the 2^−ΔΔCT^ method^44^.

### Quantification of cantharidin by GC

The quantification of cantharidin produced by the blister beetle was performed on a gas chromatography system essentially as described previously with slight modifications^22^. Cantharidin was quantified using GC-2010 Plus (SHIMADZU, Kyoto, Japan) with HP-5 capillary column (15 m × 0.53 mm × 1.5 μm) and flame ionization detector. Conditions of GC were as follows: initial temperature was 140 °C maintained for 2 min then raised at 12 °C/min increments to 240 °C maintained for 5 min; injector and detector temperatures were both 250 °C. Nitrogen was applied as a carrier gas at a constant flux of 30 mL/min, hydrogen and air at 50 ml/min and 400 ml/min, respectively. 1 μL of each sample was injected with three independent repeats. The quantification of cantharidin was performed using the standard curve for pure cantharidin with hexadecane as an endogenous control (Sigma, MO, USA).

## Electronic material

Supplementary information
