# Supplementary material for: Characterization of Juvenile Hormone Related Genes Regulating Cantharidin Biosynthesis in Epicauta chinensis
Source: Sci Rep. 2017 May 23;7:2308. doi: 10.1038/s41598-017-02393-w (PMC5442126; doi:10.1038/s41598-017-02393-w)
Supplement: Supplementary file 1 — Supplementary information [file 41598_2017_2393_MOESM1_ESM.pdf]

# Characterization of Juvenile Hormone Related Genes Regulating Cantharidin Biosynthesis in *Epicauta chinensis*

**Ming Jiang, Shumin Lü and Yalin Zhang ***

Key Laboratory of Plant Protection Resources and Pest Management, National Ministry of Education, Northwest A&F University, Yangling, Shaanxi, 712100, China
***Correspondence: yalinzh@nwsuaf.edu.cn; Tel.: +86-029-87082808

**Authors' information**

**First Author:** PHD Ming Jiang

**Address:** Key Laboratory of Plant Protection Resources and Pest Management, National Ministry of Education, College of Plant Protection, Northwest A&F University, Yangling, Shaanxi, 712100, China. Tel: (00)86-189 9184 9973

**E-mail:** mingjiang@nwsuaf.edu.cn

**Second Author:** Dr. Shumin Lü

**Address:**Key Laboratory of Plant Protection Resources and Pest Management, National Ministry of Education, College of Plant Protection, Northwest A&F University, Yangling, Shaanxi, 712100, China Tel: (00)86-158 0923 9306

**E-mail:** shuminlv@nwsuaf.edu.cn

**Corresponding Author:** Pro.Yalin Zhang

**Address:**Key Laboratory of Plant Protection Resources and Pest Management, National Ministry of Education, College of Plant Protection, Northwest A&F University, Yangling, Shaanxi, 712100, China. Tel: (00)86-29-87082808

**E-mail:** yalinzh@nwsuaf.edu.cn

*Supplementary table*

Table S1. Primers for gene clone and Real-time PCR

| Target | Genes | Primers | Sequences (5′-3′) | Tm (℃) |
|---|---|---|---|---|
| 5′&3′ RACE | MFE | MFE3RS1 | ATCGGCTCGCTACATTGTTTCCTAA | 56 |
| | | MFE3RS2 | CCGACAACATTACCGAATCGCTCCG | 60 |
| | | MFE5RA1 | ATGGGTAGAAATCTGGTTGTTGCCG | 57 |
| | | MFE5RA2 | CAATGTAGCGAGCCGATTTGGATGC | 59 |
| | JHEH | JHEH3RS1 | AACGATTCACCTGTAGGCTTAG | 53 |
| | | JHEH3RS2 | CGGATTTGGATGGTGGACATTTTGC | 57 |
| | | JHEH5RA1 | CACAGCCATTTGTACGGCACCTA | 57 |
| | | JHEH5RA2 | CGCCAATCGTACTTTGTTAGCCAG | 57 |
| | JHAMT | JHAMT3RS1 | AGAAATGGAACACCTATTGACCCGA | 56 |
| | | JHAMT3RS2 | GTCGTGAAGGCAGAATACAAATGGC | 57 |
| | | JHAMT5RA1 | GCCACCCAATGTAGACAATAAAACGAG | 58 |
| | | JHAMT5RA2 | AATACACCACCATCACCACAACCGA | 57 |
| ORF | MFE | MFE-F | GGGGGGTTTGAGTAGTTCGTAGTGG | 52 |
| | | MFE-R | TCCACCACCTTTTTATTTGAACCCA | |
| | JHEH | JHEH-F | GGTCTACTTGTGGAACCAAAAGCAAT | 55 |
| | | JHEH-R | TTTAACAAACAAAAAATCATTTCTTATT | |
| | JHAMT | JHAMT-F | GCAAAGTGGGGAAATAACGA | 55 |
| | | JHAMT-R | CCCAAAATTATGTTGTTTAAATAGAGA | |
| dsRNA | MFE | MFEr-F | TCAACACGGACACATCGAAT | 58 |
| | | MFEr-R | ACCGACTCGATATCCACCAG | |
| | JHEH | JHEHr-F | CGGCAGTTTTATACCGCAAT | 58 |
| | | JHEHr-R | CCACCATCCAAATCCGATAC | |
| | JHAMT | JHAMTr-F | TTACCGCACAATTTCAAGCA | 58 |
| | | JHAMTr-R | GTTTAGCAAACGCTGCAACA | |
| | GFP | GFPr-F | GAGAAGAACTTTTCACTGCA | 55 |
| | | GFPr-R | TGTTGATAATGGTCTGCTAG | |
| qRT-PCR | MFE | MFE-QS | CACCATTAGGAATCGCCCATAG | 60 |
| | | MFE-QA | GGACGCTGTAGAGACTGGTTAG | |
| | JHEH | JHEH-QS | ACGCGCATCTGATGTATCC | 60 |
| | | JHEH-QA | ACCAACAGTGTCCGGTTTAG | |
| | JHAMT | JHAMT-QS | TCCGTTCATCAGTCGTTTACTT | 60 |
| | | JHAMT-QA | GCCATTTGTATTCTGCCTTCAC | |
| | Actin | Ecactin-QS | TCTGGTCGTACAACTGGTATTG | 60 |
| | | Ecactin-QA | CGTAGGATAGCATGCGGTAAA | |

*Supplementary figures*

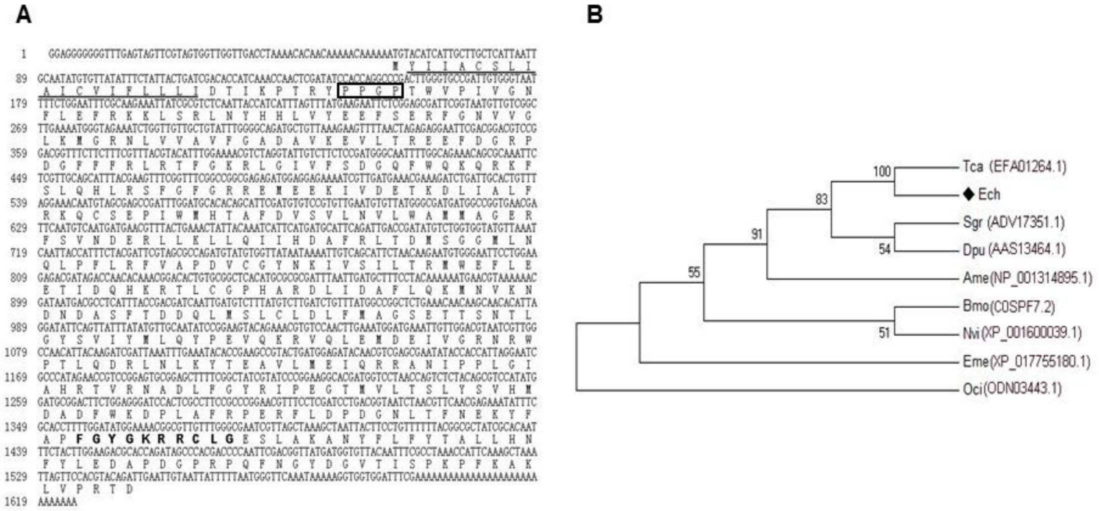


**Figure S1. Sequence analysis of *Epicauta chinensis* methyl farnesoate epoxidase.** (**A**) cDNA and deduced amino acid sequence of EcMFE. Cytochrome P450 conserved site is in bold. Transmembrane region is underlined. Microsomal P450 typical “PPGP” hinge is boxed. (**B**) Phylogenetic relationship of MFE with sequences from insects. Species abbreviations are as follows: Ame: *Apis mellifera*; Bmo: *Bombyx mori*; Dpu: *Diploptera punctate*; Eme: *Eufriesea Mexicana*; Nvi: *Nasonia vitripennis*; Oci: *Orchesella cincta*; Sgr: *Schistocerca gregaria*; Tca: *Tribolium castaneum*. GenBank number showed in parenthesis.

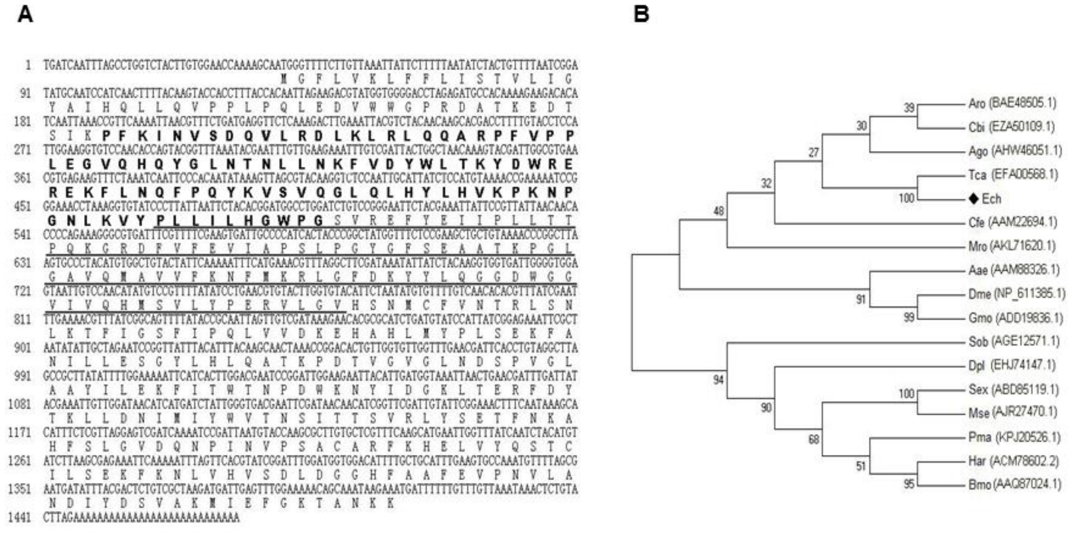


**Figure S2. Sequence analysis of *Epicauta chinensis* juvenile hormone epoxide hydrolase.** (**A**) cDNA and deduced amino acid sequence of EcJHEH. Epoxide hydrolase N-terminus domain is in bold. Alpha/beta hydrolase domain is underlined. (**B**) Phylogenetic relationship of JHEH with sequences from insects. Aae: *Aedes aegypti*; Ago: *Aphis gossypii*; Aro: *Athalia rosae*; Bmo: *Bombyx mori*; Cbi: *Cerapachys biroi*; Cfe: *Ctenocephalides felis*;

Dme: *Drosophila melanogaster;* Dpl: *Danaus plexippus*; Gmo: *Glossina morsitans morsitans*; Har: *Helicoverpa armigera*; Mro: *Macrobrachium rosenbergii*; Mse: *Mythimna separate*; Pma: *Papilio machaon*; Sex: *Spodoptera exigua*; Sob: *Spilarctia obliqua*; Tca: *Tribolium castaneum*. GenBank number showed in parenthesis.

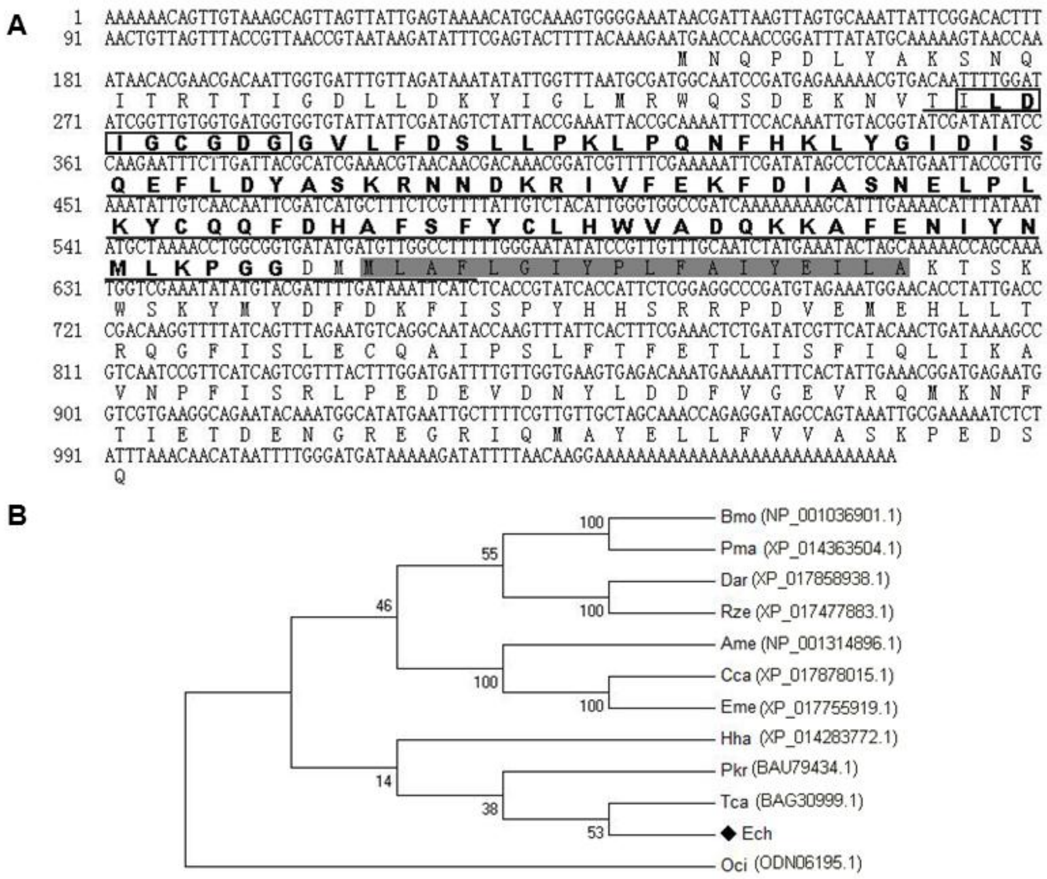


**Figure S3. Sequence analysis of *Epicauta chinensis* juvenile hormone acid O-methyltransferase.** (**A**) cDNA and deduced amino acid sequence of EcJHAMT. Methyltransferase domain is in bold. S-adenosylmethionine-dependent methyltransferases domain is underlined. Transmembrane region is marked in gray shadow. The functional motif is boxed. (**B**) Phylogenetic relationship of JHAMT with sequences from insects. Ame: *Apis mellifera*; Bmo: *Bombyx mori*; Cca: *Ceratina calcarata*; Dar: *Drosophila arizonae*; Eme: *Eufriesea Mexicana*; Hha: *Halyomorpha halys*; Oci: *Orchesella cincta*; Pkr: *Planococcus kraunhiae*; Pma: *Papilio machaon*; Rze: *Rhagoletis zephyria*; Tca: *Tribolium castaneum*. GenBank number showed in parenthesis.
